# The impact of road environments on rural periodic market travel satisfaction: a heterogeneity analysis of travel modes

**DOI:** 10.3389/fpubh.2024.1418851

**Published:** 2024-06-05

**Authors:** Hong Xu, Ping Liang, Hao Zhu, Mingyang Li, Haimei Li, Igor Martek, Yibin Ao

**Affiliations:** ^1^College of Environment and Civil Engineering, Chengdu University of Technology, Chengdu, China; ^2^Humanities and Law School, Chengdu University of Technology, Chengdu, China; ^3^College of Management Science, Chengdu University of Technology, Chengdu, China; ^4^School of Architecture and Built Environment, Deakin University, Geelong, VIC, Australia; ^5^Digital Hu Huanyong Line Research Institute, Chengdu University of Technology, Chengdu, Sichuan, China

**Keywords:** road environment, periodic market travel, heterogeneity analysis, travel modes, travel satisfaction

## Abstract

**Introduction:**

Travel satisfaction as experienced by rural residents is closely related to personal physical and mental health, as well as rural economic conditions. An improved rural road environment can be expected to enhance villagers’ satisfaction with regards to visits to markets, but to date this has not been established empirically.

**Methods:**

In this study, a questionnaire was designed to obtain local residents’ evaluations of road environment characteristics for periodic market travel. And we use an Oprobit regression model and Importance-Performance Map Analysis (IPMA) to explore the heterogeneity of the 14 key elements of the “home-to-market” road environment impact on villagers’ satisfaction under different modes of travel.

**Results:**

The results of the study reveal that villagers expressed dissatisfaction with the current lack of sidewalks and non-motorized paths, and except for road traffic disturbances and road deterioration, which did not significantly affect mode of travel, other factors proved significant. Significantly, bus services are associated with a significant positive effect on walking, non-motorized and bus travel satisfaction, while distance travel also affects walking, non-motorized and motorized travel satisfaction. It is worth noting that greening and service facilities negatively affect motorized travel satisfaction. In summary, road width, sidewalks, bus service, and road deterioration, are among the elements most in need of urgent improvement for all modes of travel.

**Discussion:**

The characteristics of the road environment that influence satisfaction with travel to the periodic market vary by travel mode, and this study is hoped to provide data support and optimization recommendations for the improvement of the rural road environment in China and other countries.

## 1 Introduction

Travel satisfaction is a subjective experience arising from the process of travel (1), and is an important indicator of locale livability (2, 3). In rural China, periodic markets serve as the primary venue for residents to procure production and livelihood materials (4), engage in the sale of agricultural and sideline products (5), as well as partake in leisure, recreational, and social activities. Periodic market travel is an important regular activity for rural residents (6). Enhancing satisfaction with periodic market visits can increase the frequency of rural residents’ visits to these markets, facilitating greater participation in market trade activities and thereby promoting rural economic development. This holds significant importance for achieving sustainable rural development (7). Engaging in market activities allows rural residents to access a more diverse range of food, ensuring nutritional balance and consequently improving their quality of life (4). Moreover, participating in market activities is beneficial for reducing mental stress, lowering the risk of chronic diseases, and positively impacting residents’ health (8, 9), thus enhancing residents’ subjective well-being (10).

Although a considerable number of scholars have worked on analyzing the factors influencing travel satisfaction, including travel mode (11, 12), travel time (13, 14), travel purpose (15), built environment (accessibility, density, convenience) (16–18), these were conducted in urban areas, and there is a paucity of research on rural travel satisfaction. Moreover, roads serve as a bridge connecting rural residents with their often frequented markets, and the quality of roads and their ancillary facilities are an important factor affecting the satisfaction of rural residents (19, 20). However, current measurements of the road environment tend to be macroscopic, with less literature considering micro-environmental elements, leading to a lack of in-depth analysis of elements such as road surface conditions, road cleanliness, roadside facilities, etc.

In addition, as a consequence of socio-economic and technological developments, the travel modes of rural residents have gradually diversified. Specifically, rural car ownership has changed significantly (21), and villagers’ expectations of the travel road environment have been raised. Thus, the relationship between rural road environment and travel mode has received attention. Li et al. (17), Nathan et al. (22) and Ao et al. (23) found that the road environment is an important influence on rural residents’ choice of travel mode. Zacharias and Liu (24) indicated that travelers’ perception of the environment and satisfaction varies by travel mode. Nevertheless, both ignored the heterogeneity of the mechanism by which the road environment influences travel satisfaction across travel modes.

Therefore, this study starts from the definition of the road environment by Fitch et al. (25), and focuses on the road environment from home to periodic market by investigating the travel mode, road environment and travel satisfaction of rural residents traveling regularly to market. The main aims of this study are as follows: (1) considering different modes of travel to the periodic market, a grouped Oprobit model was used to analyze the effect of the road environment on satisfaction. (2) An assessment was made using importance-performance analysis of the urgency of improving the environmental characteristics of rural roads across different modes of travel. (3) A synthesize of the results of the analysis is made and measures and recommendations put forward to improve the rural road environment. The aim of this research is to provide valuable insights for improving the road environment and travel satisfaction in rural areas of China and other developing countries.

## 2 Literature review

### 2.1 Periodic market travel satisfaction

Friman et al. (26) viewed travel satisfaction as a domain-specific subjective assessment of well-being. In their view, traveler satisfaction refers to the extent to which the transportation system provides services that meet the needs and services of the traveler. De Vos et al. (27) defined travel satisfaction as the relationship between general subjective satisfaction and the well-being experienced during the travel process; an emotional and subjective experience resulting from the travel process. Gao et al. (28) combined the two views and stated that travel satisfaction refers to people’s evaluation and experience of transportation services during the travel process. Satisfaction with periodic or regular travel to market is defined in this study as the comprehensive evaluation of road facilities and their services by rural residents during their commute from “home to periodic market.”

Although there is a paucity of literature examining satisfaction with trips to periodic market, there are studies that have investigated the factors that influence periodic market participation and periodic market access. Topçu (29) argues that the distance to market affects the frequency of periodic market travel. Aram et al. (30) suggests that green spaces are significant factors influencing the frequency of market visits and participation in market activities. A number of transportation-related factors can also affect residents’ travel responses. Such factors being unsuitable or inadequate public transportation services (31), and imperfect transportation infrastructure (32). Inadequate road networks and poor road conditions can also act as barriers to marketplace access for rural residents (33). Liu et al. (34) investigated the factors influencing rural residents’ access to periodic markets from a geospatial perspective and showed that proximity of town centers, and stores around village centers had a significant effect on access. In addition, some village markets are set up along the roadside of transit highways. Consequently, an increase in the number of motor vehicles in town can be expected to interfere with traffic and pedestrian flow. Moreover, mobile vendors occupying roadside zones leads to congestion of vehicular traffic, while the increased influx of residents crossing transit highways in the pursuit of shopping activities results in increased frequency of traffic accidents and pedestrian collisions (35).

### 2.2 Mode of travel and travel satisfaction

There are differences in travel satisfaction ratings by travel mode. For example, several studies consistently indicate that the use of public transportation for travel is associated with low levels of travel satisfaction (11, 36–38). Satisfaction with public transportation travel was found to be higher than that for car or walking with regard to school children (39). Lunke (40) further conducted a study on the satisfaction of traveling by different modes of public transport and found that commuting by train and metro was more satisfying than commuting by bus or tram. For active travel modes, walking appears more satisfying than non-motorized trips; when using motorized vehicles, company shuttle commuting is preferred, followed by car (12, 14). Yet another study stated that there is no significant correlation between travel mode and travel satisfaction (15). Taking a general look at the established research on rural periodic market travel, no research literature has explored the satisfaction of rural residents regarding periodic market travel, based on different modes of travel.

### 2.3 Road environment and travel satisfaction

A growing body of research has focused on the relationship between road environment and travel satisfaction. Specifically, accessibility and walkability among the 5D variables of the built environment were found to be important factors in travel satisfaction (16, 41–43). From a micro perspective, Li et al. (17) argue that the provision of sidewalks, bike lanes, and highways has a positive effect on travel satisfaction, either directly or indirectly. Studies by van den Berg et al. and Xu et al. (39, 44), also show the impact of non-motorized lane settings on travel satisfaction. Wu et al. (45) suggests that insufficient pedestrian infrastructure, high volumes of vehicular traffic, excessive on-street parking, or illegal parking collectively diminish pedestrian satisfaction. Jung et al. (46) found that increase in the number of lanes, and installation of public transit and pedestrian lanes affect pedestrian satisfaction.

The impact of road conditions on travel satisfaction has also been considered. Ye and Titheridge (38) and Majumdar et al. (36) found that traffic congestion is associated with low levels of travel satisfaction. Moreover, the street leveling will positively affect walking satisfaction (47). In addition, Han et al. (48) found that esthetic features such as roadway landscaping and cleanliness affect recreational travel satisfaction. Ta et al. (15) also shown that the presence of trees and exposure to green space positively affects travel satisfaction. In terms of transportation systems and services, factors such as traffic safety signs, traffic signals, and speed limits, significantly affect cycling satisfaction (49). Majumdar et al. (36) noted that bus stop safety, and bus stop accessibility, also improve the travel satisfaction of commuters. Public spaces are also crucial factors influencing travel satisfaction. For instance, Ma et al. (50) concluded that recreational facilities (benches, shade, etc.) have a significant effect on walking satisfaction. Furthermore, Fan et al. (51) explored the impact of service facilities on satisfaction with barrier-free travel.

In addition to the direct effects of road environment on travel satisfaction, Ye and Titheridge (52) stated that the effect of the built environment on travel satisfaction are indirect, but nevertheless highly influenced by travel mode choice. There are differences in the impact of road environment factors on travel satisfaction across travel modes. For example, in regards to walking, green space exposure during travel has a strong positive effect on travel satisfaction; though there was no significant correlation for cycling or e-cycling modes (15). Zacharias and Liu (24) conducted a study on the factors influencing the access to metro stations across different modes, finding that road connectivity, intersection safety, greenery, and shade, are positive factors for walking mode; service facilities, parking facilities, detours, and distance affect riding mode; while for transit mode, it is bus stop location that most impacts satisfaction. Nevertheless, current research on rural travel satisfaction has yet to examine micro-level environmental factors.

### 2.4 Contribution of the literature to this study

A review of the existing literature thus reveals significant limitations of previous studies. These are: (1) Research on travel satisfaction is limited in its focus to urban areas, whereas studies on rural travel satisfaction have been neglected. This is an important omission, as the unique characteristics of rural areas indicate different conclusions may arise. (2) Existing research has explored factors influencing general travel satisfaction, commuting satisfaction, and satisfaction with specific modes of travel. However, there is a lack of research on the factors influencing satisfaction with specific activities such as visits to rural periodic markets. (3) Regarding the factors influencing travel satisfaction, scholars tend to focus more on macro-level road environment elements, overlooking micro-level environmental factors. (4) There is limited research exploring the direct or indirect impact of road environment on travel satisfaction for different travel modes. Comparative analyses regarding differences in satisfaction across different modes of travel are thus lacking.

To address the limitations, a thorough analysis of rural periodic market travel satisfaction across different travel modes is warranted. This study focuses on rural areas in Chengdu, Sichuan, China. (1) It constructs a system of rural road environment elements from a micro-level perspective, exploring residents’ satisfaction with road environment elements across different travel modes. (2) Utilizing the Oprobit regression model as a quantitative research method, it analyzes the heterogeneity of the impact of road environment elements on periodic market travel satisfaction under those different modes. (3) Combining IPA analysis, it offers recommendations for road environment improvements in order to enhance periodic market travel satisfaction, and moreover provides insights that relevant authorities may avail themselves of in order to improve rural travel environments and more effectively plan rural development.

## 3 Methodology

### 3.1 Questionnaire design

To achieve the research objectives outlined above, a questionnaire was developed for this study, comprising four sections, as outlined below:

1. Individual demographic characteristics of rural residents, such as age, gender, education level, occupation, household income, and family structure.
2. Travel characteristics, including frequency of periodic market visits, mode of travel for periodic market visits, time required from home to the periodic market, and purposes of periodic market visits. The modes of travel for periodic market visits are categorized as walking, non-motorized vehicles (including bicycles and electric bikes), motorized vehicles (including motorcycles, cars, and agricultural vehicles), and bus.
3. Rural residents’ evaluation of the importance of road environment characteristics for periodic market travel.
4. Rural residents’ satisfaction with road environment characteristics for market travel and overall satisfaction with periodic market travel.

Drawing from existing literature, this study considers 14 road environment characteristics: traffic safety signs (49), road width (46), sidewalks (17), non-motorized vehicle lanes (39), pavement materials (33), disturbance situation (35), road deterioration (47), recreational facilities (50), service facilities (34), cleanliness (48), road greening (15), parking areas (24), bus services (38), and travel distance (53). During the survey process, a 5-point Likert scale was used to rate the road environment characteristics based on the perceived importance and satisfaction of rural residents: 1, indicating very unimportant or very dissatisfied, to 5, indicating very important or very satisfied.

### 3.2 Data sources

According to the latest Chengdu City Census Report, the proportion of permanent residents in Xindu District and Shuangliu District ranks highest in respect of the permanent population of Chengdu City. Additionally, within the administrative area of Chengdu City, the proportion of permanent rural residents in Xindu District and Shuangliu District ranks among the top three in terms of the total rural population. Of the county-level cities in Chengdu, Jianyang City, has a moderate proportion of rural inhabitants compared to the total rural population, but it is the only county-level city in Chengdu City that was recognized as an advanced area for rural revitalization in 2021. For this study, Xindu District, Shuangliu District, and Jianyang City in Chengdu City are selected as the research catchments, with villages randomly chosen for the survey.

In July 2022, the research team conducted a small-scale pre-survey to ensure the effectiveness and comprehensibility of the questionnaire results. Based on the findings from the preliminary household visits, modifications were made to the questionnaire content and language to better align with rural realities and enhance the questionnaire’s comprehensibility. Prior to the official survey, a workshop was held to explain the questionnaire content, provide training to surveyors, and refine the questionnaire to ensure readability. In October 2022, the research team dispatched 16 surveyors divided into 3 groups to conduct household surveys in Xindu District, Shuangliu District, and Jianyang City. Surveyors centered their activities around village committees or community centers, oportunistically soliciting villagers for the surveys. When administering the questionnaire, surveyors first explained the purpose of the survey to the respondents and then invited them to participate. Respondents’ voluntary participation was confirmed, and anonymity assured. Surveyors remained nearby to clarify any questions and assist respondents in completing the questionnaire. A total of 408 questionnaires were collected during the survey, with 387 deemed valid, resulting in an effective rate of 94.8%.

### 3.3 Data analysis

#### 3.3.1 Ordered probit (Oprobit) regression model

To compare the differences in the impact of road environment elements on periodic market travel satisfaction under different travel modes, this study employed the Oprobit regression model to evaluate the differences in perceived satisfaction under different travel modes. The constructed Oprobit model is as follows:


(1) $$y_{i}^{*}=\alpha_{1}+\beta_{i1}x_{i1}+\ldots +\beta_{ik}x_{ik}+\varepsilon_{i}$$

In Equation (1), $\alpha_{1}$ represents the intercept term, $\beta_{i}$ represents the coefficients to be estimated, $x_{i}$ represents explanatory variables and control variables, $\varepsilon_{i}$ represents the error term, k represents the number of explanatory variables and control variables, and $y_{i}^{*}$ represents the latent variable of residents’ periodic market travel satisfaction. $y_{i}$ represents residents’ periodic market travel satisfaction, and its relationship with $y_{i}^{*}$ is expressed as:


(2) $$y_{i}=\{\begin{array}{c} 1,\;y_{i}^{*}\leq K_{1}\;\\ 2,\;K_{1}<y_{i}^{*}\leq K_{2}\\ \ldots \\ 5,\;K_{4}<y_{i}^{*}\; \end{array}$$

In Equation (2), when $y_{i}^{*}\leq K_{1}$ , residents’ periodic market travel satisfaction is at a level of very dissatisfied; when $K_{1}<y_{i}^{*}\leq K_{2}$ , residents’ periodic market travel satisfaction is at a level of dissatisfied; and so forth, until when $K_{4}<y_{i}^{*}$ , residents’ periodic market travel satisfaction is at a level of very satisfied.

#### 3.3.2 Importance performance map analysis

IPA is a method abbreviated for Importance-Performance Analysis, where Importance refers to the significance users attach to characteristics like the environment, or the importance of characteristics to users. Performance denotes satisfaction, which is a multidimensional concept believed to be the result of comparing expectations with actual experiences. The IPA evaluation system quantifies the gap between users’ actual perceptions and expectations for various indicators, analyzing object characteristics from the dimensions of importance and satisfaction. By using a matrix consisting of four quadrants, IPA determines priorities, reflecting improvement strategies and implementation sequences.

In this study, based on the IPA analysis method, the Importance Performance Map Analysis (IPMA) was constructed to provide suggestions for improving rural residents’ market travel satisfaction.

## 4 Results

### 4.1 Descriptive statistics

#### 4.1.1 Individual characteristics of different travel modes

Table 1 presents the demographic characteristics of rural residents under different periodic market travel modes. Of the 387 respondents, the highest proportion, at 40.5%, chose non-motorized vehicle for travel, followed by those opting for motorized transportation, constituting 33.1% of the total. Additionally, 70 respondents chose bus for their travel needs, with the majority being individuals aged 60 and above. Among female respondents, the majority opted for non-motorized vehicle travel, while among male respondents, the majority opted for motorized vehicle travel. Regardless of gender, walking was the least preferred mode of travel. The respondents’ ages were generally 40 years and above, with the highest number of respondents aged over 60 years opting for bus travel. Among respondents aged below 60 years old, non-motorized vehicle travel was the most popular option. Regardless of marital and employment status, non-motorized vehicle travel was the most preferred mode, while walking was the least preferred.

**Table 1:** Descriptive statistics of the participants.

Variables	Categorizations	Periodic market travel modes				Total
		Walking	Non-motorized vehicle	Motorized vehicle	Bus	
Genders	Female	24 (6.2%)	99 (25.6%)	54 (14.0%)	50 (12.9%)	227 (58.7%)
	Male	8 (2.1%)	58 (15.0%)	74 (19.1%)	20 (5.2%)	160 (41.3%)
Age	Below 40	4 (1.0%)	40 (10.3%)	38 (9.8%)	1 (0.3%)	83 (21.4%)
	40–59	11 (2.8%)	70 (18.1%)	56 (14.5%)	16 (4.1%)	153 (39.5%)
	60 and above	17 (4.4%)	47 (12.1%)	34 (8.8%)	53 (13.7%)	151 (39.0%)
Marital status	Spouse-less	17 (4.4%)	44 (11.4%)	23 (5.9%)	39 (10.1%)	123 (31.8%)
	Spousal	15 (3.9%)	113 (29.2%)	105 (27.1%)	31 (8.0%)	264 (68.2%)
Educational level	Uneducated	9 (2.3%)	10 (2.6%)	5 (1.3%)	18 (4.7%)	42 (10.9%)
	Primary school	14 (3.6%)	66 (17.1%)	32 (8.3%)	37 (9.6%)	149 (38.5%)
	Middle school	7 (1.8%)	59 (15.2%)	35 (9.0%)	11 (2.8%)	112 (28.9%)
	Senior high school	2 (0.5%)	17 (4.4%)	48 (12.4%)	3 (0.8%)	70 (18.1%)
	University and higher	0 (0.0%)	5 (1.3%)	8 (2.1%)	1 (0.3%)	14 (3.6%)
Employment status	Unemployed	14 (3.6%)	44 (11.4%)	16 (4.1%)	42 (10.9%)	116 (30.0%)
	Employed	18 (4.7%)	113 (29.2%)	112 (28.9%)	28 (7.2%)	271 (70.0%)
Monthly household income	RMB 2,000 and below	9 (2.3%)	14 (3.6%)	5 (1.3%)	22 (5.7%)	50 (12.9%)
	RMB 2,000–5,000	13 (3.4%)	53 (13.7%)	29 (7.5%)	20 (5.2%)	115 (29.7%)
	Over RMB 5,000	10 (2.6%)	90 (23.3%)	94 (24.3%)	28 (7.2%)	222 (57.4%)
Time from home to periodic market	10 min or less	2 (0.5%)	26 (6.7%)	42 (10.9%)	3 (0.8%)	73 (18.9%)
	10–20	7 (1.8%)	115 (29.7%)	82 (21.2%)	29 (7.5%)	233 (60.2%)
	20–30	7 (1.8%)	14 (3.6%)	4 (1.0%)	24 (6.2%)	49 (12.7%)
	Over 30 min	16 (4.1%)	2 (0.5%)	0 (0.0%)	14 (3.6%)	32 (8.3%)
Purpose of the fair	Non-shopping	1 (0.3%)	10 (2.6%)	4 (1.0%)	9 (2.3%)	24 (6.2%)
	Shopping	31 (0.8%)	147 (38.0%)	124 (32.0%)	61 (15.8%)	363 (93.8%)
Total		32 (8.3%)	157 (40.5%)	128 (33.1%)	70 (18.1%)	387

Regarding education level, a significant portion had received primary and middle school education, with both groups favoring non-motorized vehicle travel. Those with no formal education tended to prefer bus travel. Concerning monthly household income, nearly 60% earned over RMB 5,000 per month, and they held a preference for motorized vehicle travel. Those earning between RMB 2,000–5,000 per month favored non-motorized vehicle travel. Those earning below RMB 2,000 per month tended to rely on bus travel.

In terms of travel time from home to the periodic market, 60.2% of respondents indicated a duration of 10–20 min, mainly through non-motorized and motorized vehicle modes. Busses averaged 20–30 min, while walking typically took over 30 min. Regarding the purpose of travel to markets, almost all respondents visited in order to purchase daily necessities (93.8%), with a small percentage attending for recreational purposes, or to sell agricultural products.

#### 4.1.2 Road environment perception under different travel modes

**Figure 1:**
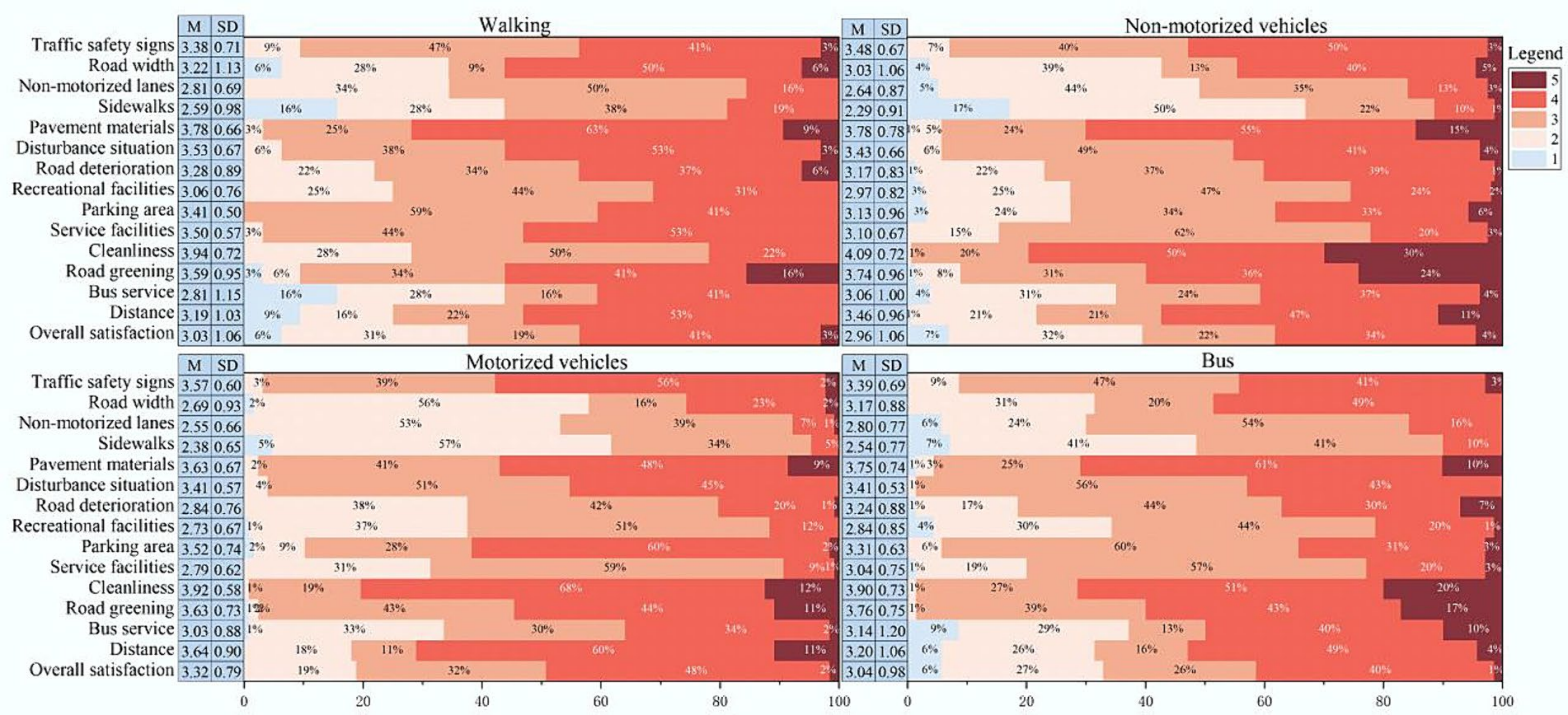
Descriptive statistics of residents’ perception of satisfaction.

Regarding the different modes of market travel for rural residents, Figure 1 depicts their satisfaction with various road environment elements and overall satisfaction.

For rural residents who travel to market on foot, the highest satisfaction rating is for road cleanliness (*M* = 3.94), followed by pavement materials (*M* = 3.78). Conversely, sidewalks receiving the lowest rating, with 44% of respondents expressing dissatisfaction, and no respondents indicating strong satisfaction. The overall satisfaction score for the road environment is 3.03, indicating an average level of satisfaction.

For residents who travel to market by non-motorized vehicles, road cleanliness (*M* = 4.09) receives the highest rating, with 30% of respondents expressing extreme satisfaction. This is followed by pavement materials (*M* = 3.78) and road greening (*M* = 3.74). Sidewalks (*M* = 2.29) and non-motorized vehicle lanes (*M* = 2.64) are similarly rated lowest in terms of satisfaction, with 17% of residents expressing extreme dissatisfaction with sidewalk arrangements. The overall satisfaction indicates a level below average.

When using motorized vehicles for periodic market travel, villagers’ evaluation of road cleanliness (*M* = 3.92) remains the highest. Satisfaction ratings for sidewalks (*M* = 2.38) is the lowest, with over 50% of residents expressing dissatisfaction or extreme dissatisfaction. In terms of overall satisfaction, no residents chose extreme dissatisfaction, with 50% expressing satisfaction or extreme satisfaction.

For villagers using busses to travel to the periodic market, road cleanliness (*M* = 3.90) scored the highest satisfaction, followed by road greening (*M* = 3.76) and pavement materials (*M* = 3.75). Satisfaction ratings for sidewalks (*M* = 2.54) and non-motorized vehicle lanes (*M* = 2.80) are the lowest, with satisfaction for recreational facilities (*M* = 2.84) also below 3. Satisfaction scores for other elements range between 3 and 3.5, with 9% of bus users expressing extreme dissatisfaction with bus services. The overall satisfaction score is 3.04, indicating an average level of satisfaction.

Comparing the four different modes of travel, villagers’ evaluation of road cleanliness ranked consistently the highest, while sidewalks and non-motorized vehicle lanes receive the lowest ratings, with sidewalks being the least satisfactory. Overall, residents using motorized vehicles for periodic market travel have the highest overall evaluation score, while non-motorized vehicle travel has the lowest score.

### 4.2 Heterogeneity analysis of the impact of road environment on periodic market satisfaction under different travel modes

**Figure 2:**
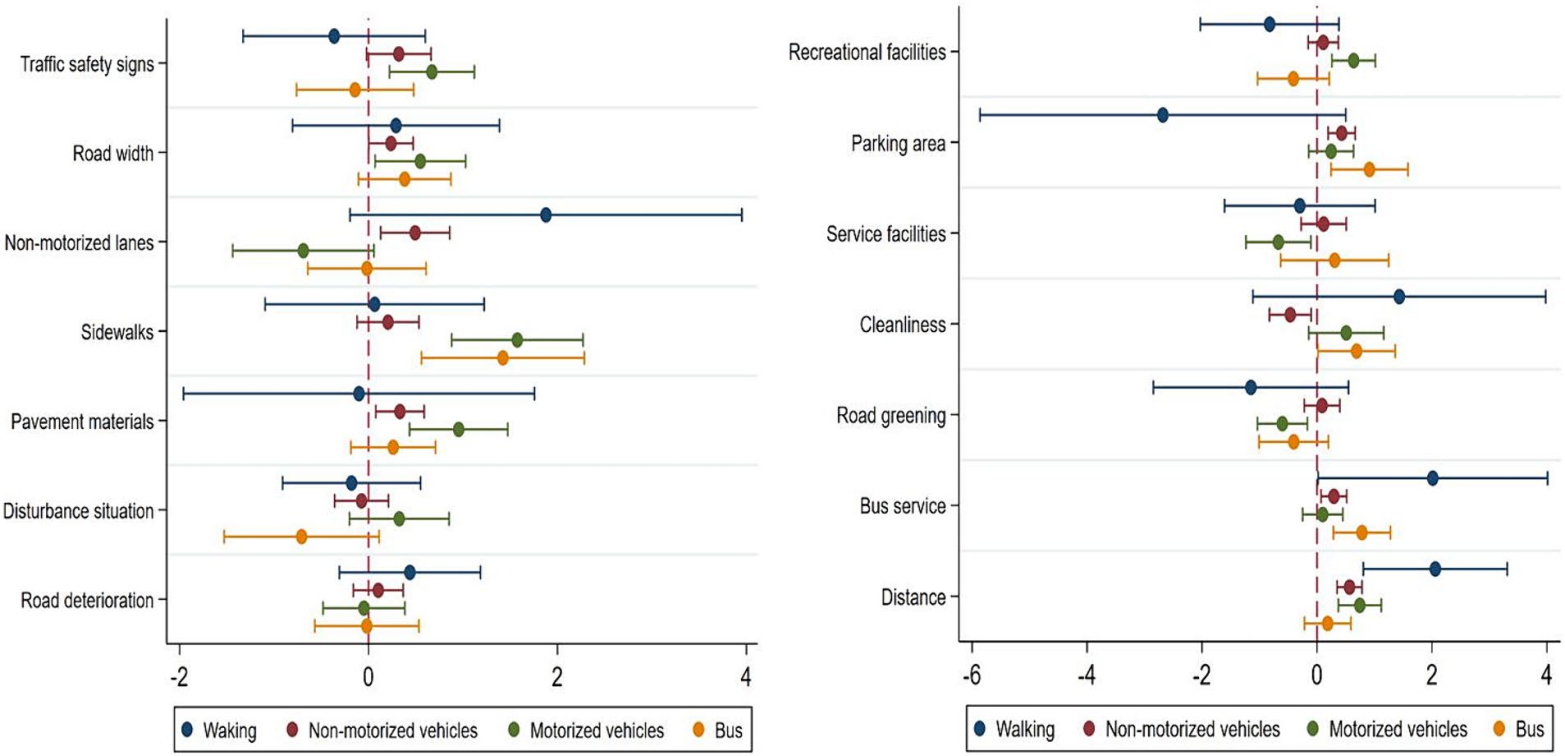
Heterogeneity analysis of different travel modes.

The influence of road environment elements on periodic market travel satisfaction varies across different modes of travel (See Figure 2).

The results indicate that the impact of various road environment elements on periodic market travel satisfaction varies to different extents across the four travel modes. Firstly, for residents who choose to walk to market, satisfaction with distance to market and bus services positively affects pedestrian travel satisfaction. Secondly, for residents who choose non-motorized vehicles as their means of transport to market, travel satisfaction is significantly positively influenced by non-motorized vehicle lanes, road width, pavement materials, distance to the market, bus services, and parking areas. By contrast, road cleanliness significantly negatively affects travel satisfaction for non-motorized vehicle travel. For residents who choose motorized vehicles for periodic market travel, traffic safety signs, sidewalks, road width, pavement materials, distance to the market, and recreational facilities all have a significant positive impact on travel satisfaction. Additionally, it is noteworthy that road greening and service facilities only have a significant impact on travel satisfaction for motorized vehicle periodic market travel, and that this impact is negative. Lastly, sidewalks, bus services, road cleanliness, and parking areas all have a significant positive impact on the travel satisfaction of residents who choose busses as their means of transport to market.

### 4.3 Importance performance map analysis

**Figure 3:**
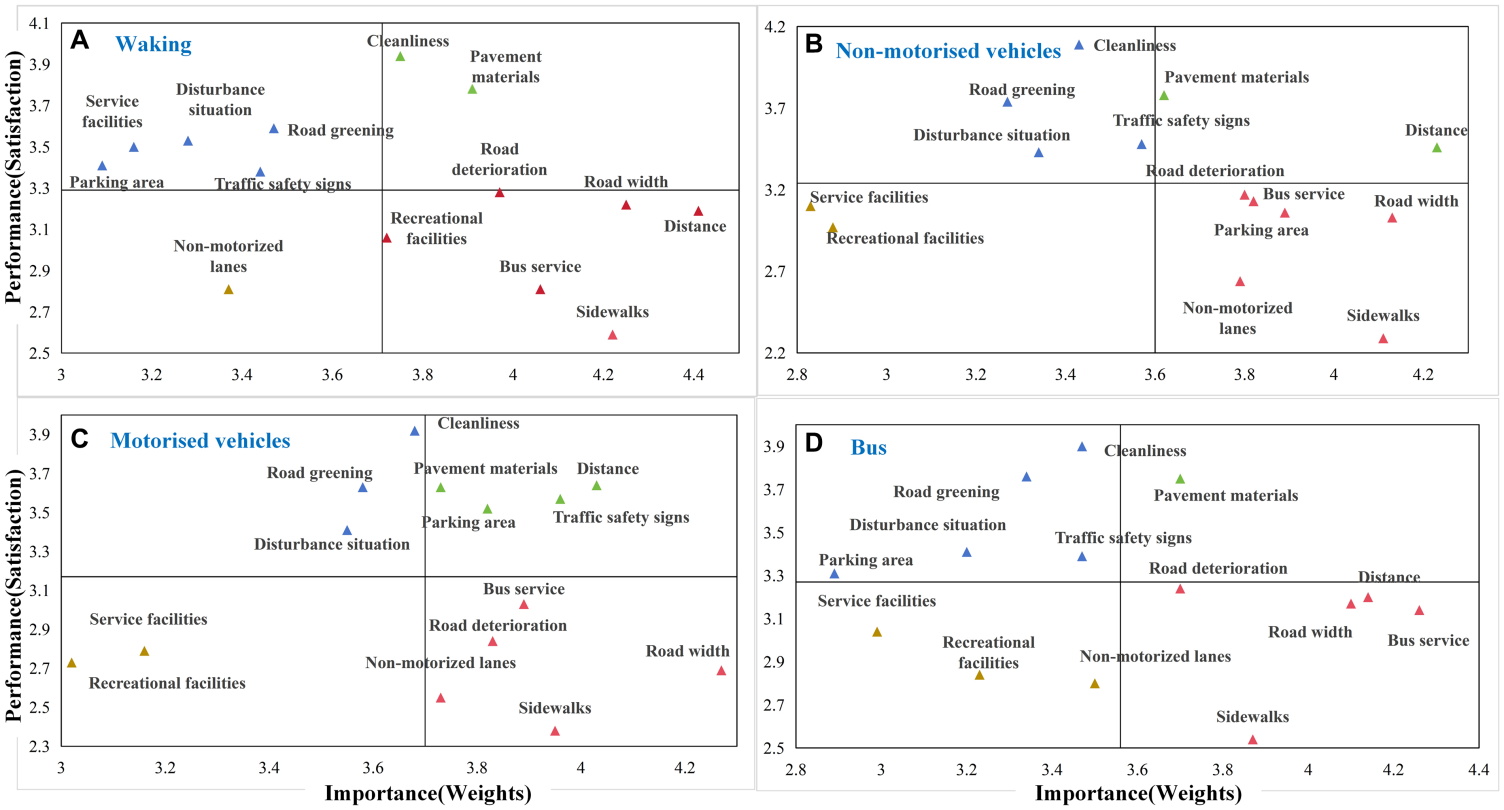
Importance performance map analysis.

In order to identify those elements needing improvement that would lift overall periodic market travel satisfaction, this study conducted an Importance Performance Analysis (IPA). Further to evaluating satisfaction across 14 road environment elements, respondents also assessed the importance of those elements. Across the different travel modes groups, the overall average satisfaction and importance of the 14 elements were plotted and partitioned into four quadrants. The Importance Performance Map Analysis (IPMA) was determined by calculating the average values of importance and satisfaction for each element (see Figure 3).

Figure 3A shows the IPMA results for the group of residents who walk to the periodic market. Among the 14 road environment elements, road cleanliness and pavement materials appear in the first quadrant, indicating relatively high importance and satisfaction. These aspects are currently in relatively good condition and need to be maintained in the future. The second quadrant includes road greening, traffic disturbances, service facilities, parking areas, and traffic safety signs. Here, satisfaction is relatively high while importance is low, suggesting that efforts to improve these aspects can be shifted to other areas needing more urgent improvement. The third quadrant consists only of non-motorized vehicle lanes, where both importance and satisfaction are relatively low, indicating no need for intervention regarding non-motorized vehicle lanes for the group of residents who walk to the periodic market. Road deterioration, recreational facilities, road width, distance to market, bus services, and sidewalks are located in the fourth quadrant, indicating high importance but relatively low satisfaction. Elements in this quadrant are in urgent need of improvement and ought to be prioritized for attention.

For rural residents who use non-motorized vehicles to travel to periodic market (see Figure 3B), no additional efforts are needed to improve distance to the market, pavement materials, service facilities, and recreational facilities. Additionally, efforts to improve road cleanliness, greening, traffic disturbances, and traffic safety signs, can be redirected to road deterioration, bus services, road width, parking areas, non-motorized vehicle lanes, and sidewalks. As a matter of comparison, for those residents who use motorized vehicles to travel to the market (see Figure 3C), parking areas appear in the first quadrant, indicating no need for urgent improvement.

Figure 3D reflects the importance-satisfaction results for residents who travel to the periodic market by bus. Only pavement materials appear in the first quadrant, indicating the need to maintain the current situation. No additional efforts are needed to improve recreational and service facilities, as well as non-motorized vehicle lanes for bus travel. Additionally, apart from sidewalks, road deterioration, road width, and bus services, distance traveled to the market also warrants timely improvement.

In summary, sidewalks, road deterioration, road width, and bus services are all located in the fourth quadrant, regardless of the travel mode. This reveals an urgent need to invest greater effort in improving periodic market travel satisfaction across all travel groups. Additionally, distance to market is problematic for both pedestrian and bus traveler groups, while residents using non-motorized and motorized vehicles seek improvements in non-motorized vehicle lanes. Furthermore, recreational facilities need improvement for residents who walk to the periodic market, while residents using non-motorized vehicles would benefit from improved parking facilities.

## 5 Discussion

### 5.1 Perceived heterogeneity across travel modes

Regardless of travel mode chosen, the satisfaction ratings for road cleanliness, greening, pavement materials, and road disruptions, are relatively high. This suggests that the road cleanliness and greening conditions are acceptable during rural residents’ periodic market travel, and that the pavement materials contribute to the overall desirability of travel, with minimal occurrences of vendors occupying roads or vehicles obstructing them. Regression results show that road disturbances and road deterioration do not significantly affect the overall satisfaction of periodic market travel. However, other road environment factors exhibit varying impacts on travel satisfaction across different travel modes.

With respect to sidewalks, satisfaction ratings are the lowest across the four travel modes. However, sidewalks significantly positively influence the satisfaction of periodic market travel for both motor vehicles and busses. The proper installation of pedestrian sidewalks can reduce conflicts between motorized vehicles, busses, and pedestrians, enhancing safety and convenience, thus increasing their satisfaction. It is worth noting that the regression results show that the impact of sidewalks on pedestrian travel satisfaction is not significant, which differs from the findings of Jung et al. and Kim et al. (46, 47). This may be because the space between roads and buildings as well as the shoulders between roads and fields are wide enough to provide pedestrians with greater space, while rural road traffic is also relatively low.

Additionally, results indicate that the impact of sidewalks on non-motorized vehicle travel satisfaction is not significant, whereas Tran et al. (54), suggest that sidewalks are an important factor influencing rideability. Based on the actual situation in rural areas, rural residents are aware of the safety issues associated with the blending of pedestrians and non-motorized vehicles. Still, due to the limited width of rural roads, the coexistence of pedestrians and vehicles is a common phenomenon. Residents indicate that they can accept pedestrians and non-motorized vehicles coexisting. However, they still assert the importance of setting up sidewalks for periodic market travel when road conditions permit (as shown in Figure 3B).

Rural residents have given relatively low satisfaction ratings for non-motorized vehicle lanes. However, improving the setup of non-motorized vehicle lanes only significantly impacts the satisfaction of non-motorized vehicle travel. The existence of non-motorized vehicle lanes can separate bicycles or electric vehicles from other motorized traffic, thereby enhancing the safety of cyclists (54). In the absence of non-motorized vehicle lanes, as mentioned earlier, pedestrians can choose to walk on raised embankments or road shoulders. For villagers traveling by motor vehicles and busses, on one hand, their size, speed, and safety, are seen positively. On the other hand, allocating space for non-motorized vehicle lanes on existing rural roads would reduce available space for motorized vehicle lanes.

Traffic safety signs, recreational facilities, convenience service facilities, and road greening only have a significant impact on motorized vehicle travel. Interestingly, the research results indicate that convenience service facilities have a significant negative impact on the satisfaction of motorized vehicle travel. This may be because the presence of convenience facilities attracts more people, while rural roadside convenience facilities (such as restrooms, convenience stores, etc.) typically lack parking areas. This leads to traffic congestion and disruptions, thus reducing travel satisfaction. Additionally, road greening also has a significant negative impact on motorized vehicle travel satisfaction. This finding contradicts some previous research results that suggest that good road greening can improve travel satisfaction (48, 55). By contrast, this finding confirms the research results of Zhu et al. (56), who suggest that the canopy of urban roadside trees are too large, their spacing being too wide or too narrow, and their height being too low such as to obstruct the sightlines of motorized vehicle users. Similarly, in rural areas, road greening can affect the visibility of traffic signs and signals. Moreover, under wet conditions, road greening may make the road surface more slippery or uneven, leading to decreased vehicle maneuverability.

Road width and pavement material significantly positively influence the satisfaction of both non-motorized and motorized vehicle travel, while their impact on pedestrian and bus travel satisfaction is not significant. Narrow roads not only cause inconvenience in passing and overtaking but also affect the delineation of traffic functional zones such as non-motorized vehicle lanes and motorized vehicle lanes (57). Increasing road width can significantly enhance the travel satisfaction of drivers. Additionally, poor road surface materials can lead to road potholes, mud, and other detrimental conditions (58), severely affecting the satisfaction of both non-motorized and motorized vehicle travel. For pedestrians, there are more options available which can mitigate the negative effects of insufficient road width and poor road surfaces. For bus travelers, as they are not directly involved in driving, they do not need to focus on the road and traffic conditions, as cyclists and drivers do.

Parking areas and road cleanliness have a significant impact on non-motorized and bus travel, but road cleanliness has a negative effect on the satisfaction of non-motorized vehicle travel. Although there is currently no research indicating the relationship between road cleanliness and non-motorized vehicle travel, some relationships can be inferred. In terms of road cleanliness, villagers generally express high satisfaction as main roads are typically maintained. However, main roads often experience higher traffic volume or speeds, which may result in more exhaust emissions and noise. Additionally, rural roads are generally narrow and lack designated lanes for non-motorized vehicles, thus negatively impacting the experience and safety of cyclists. This study found that parking areas have an impact on non-motorized vehicle travel satisfaction, which is also emphasized in the research by Zhan et al. (59) and Zacharias and Liu (24). With the increasing number of electric vehicles in rural areas, more residents are choosing electric vehicles as their mode of travel to markets. However, there are few dedicated parking lots near periodic markets, leading to an increasing parking problem. According to surveys, most vehicles are currently parked directly on the roadside, affecting pedestrian and vehicular traffic, and some residents expressed concerns about the risk of vehicle theft.

Bus services have no significant impact on motorized vehicle travel, which is consistent with the findings of Ye and Titheridge (38). The impact of bus services on bus travelers is evident (60, 61), but its effects on walking and non-motorized vehicles may be because the presence of bus services can provide alternative modes of travel for pedestrians and non-motorized vehicle users, especially during inclement weather conditions. Compared to walking, non-motorized vehicles, and motorized vehicles, the satisfaction of bus travel is less affected by distance because bus travelers prioritize factors such as frequency of service and direct routes (60, 61).

### 5.2 Capacity building of periodic market travel satisfaction

**Figure 4:**
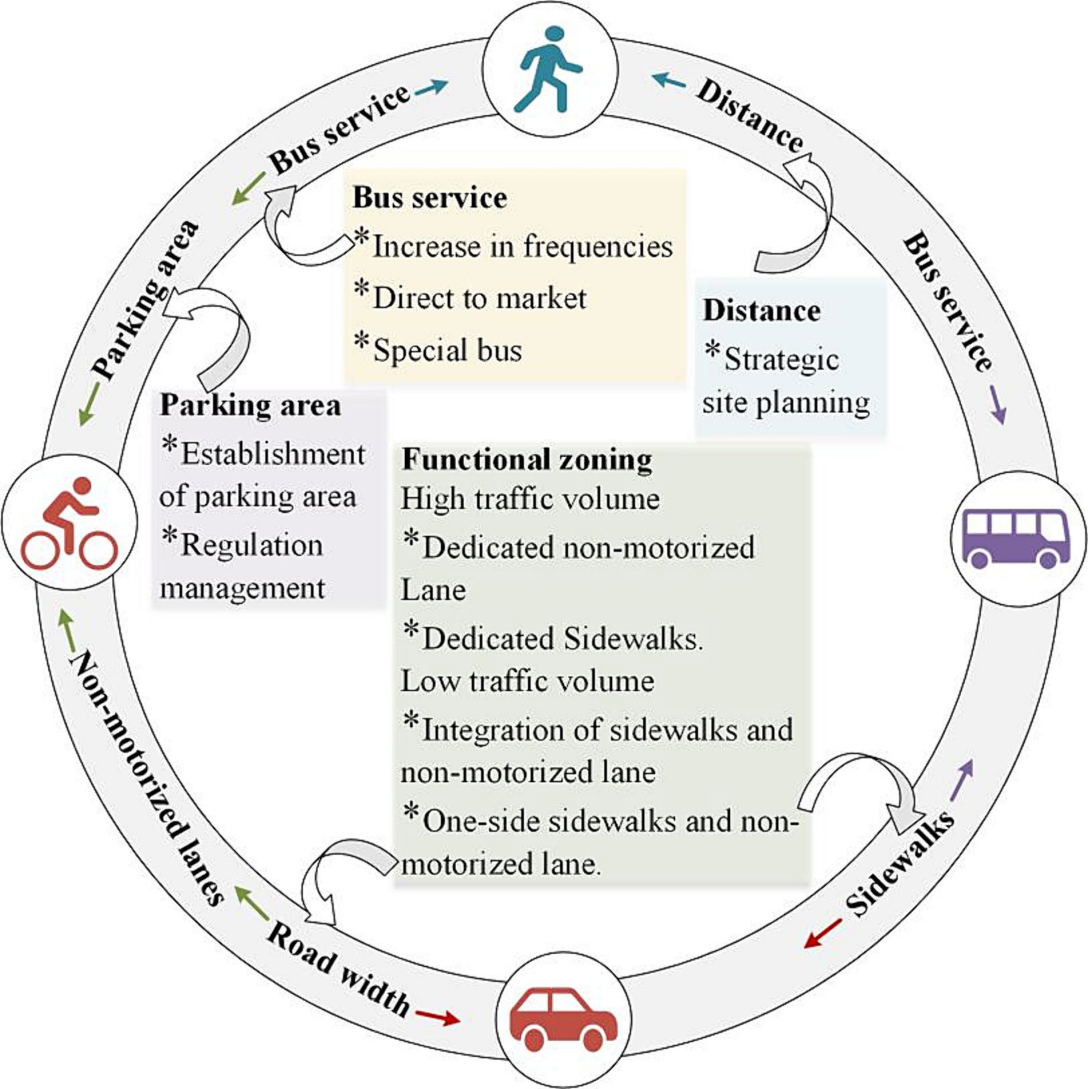
Capacity building diagram.

The results of the IPMA can be used to identify elements that need to be urgently improved in order to increase the level of satisfaction of rural residents traveling to periodic markets. The combined regression results (Figure 2) and IPMA results (Figure 3) show that bus services and travel distance are important improvement factors for residents attending periodic market on foot. In order to improve the satisfaction of non-motorized residents traveling to the periodic market, four elements can be considered: non-motorized lane provision, road width, bus services, and parking facilities. From the perspective of driving motorized residents, sidewalks and road width could be further upgraded. Improvements in sidewalks and bus services can increase bus travel satisfaction. Therefore, combining the results of this study and rural realities, the following capacity building is proposed (See Figure 4).

#### 5.2.1 Improving satisfaction with non-motorized travel is a top priority

Based on the current rural travel situation, Table 1 indicates a preference among rural residents for non-motorized vehicles, with the highest number of people choosing non-motorized vehicles for periodic market travel. The results from Figure 1 suggest that the current rural periodic market road conditions are unfavorable for non-motorized vehicle travel, with the overall satisfaction score being the lowest among the four modes of travel. Therefore, it is necessary to focus on creating a favorable environment for non-motorized vehicle periodic market travel, which would not only improve satisfaction with non-motorized vehicle travel but also promote low-carbon travel. The primary issue to address is the division of traffic functional zones. This is due to inadequate overall road width needed to support the establishment of complete functional zones. Therefore, there is a need to increase investment in widening roads to meet the requirements of setting up dual lanes, non-motorized vehicle lanes, and sidewalks on main roads. Secondly, for villages with low traffic volume, it may be appropriate to reduce the setting of functional zones, such as merging sidewalks and non-motorized vehicle lanes for combined use, or only setting sidewalks and non-motorized vehicle lanes to one side. Finally, it is worth considering incorporating the construction of parking lots into periodic market construction upgrades in order to enhance travel satisfaction and achieve standardized management of periodic market vehicles.

#### 5.2.2 Improving bus service to meet the needs of older adult individuals traveling to periodic market

Table 1 illustrates the demand of older adult individuals for periodic market travels via bus. Considering the aging population and the promotion of low-carbon travel in rural areas, enhancing bus services also represents a breakthrough in improving satisfaction with periodic market travel among rural residents. Firstly, for rural residents, due to the scattered distribution of their residences and limited destinations for daily travel, the operational hours of rural bus services remain limited, while the frequency of operation is low. Providing top-notch bus services in rural areas generally comes with high costs (62). In order to offer higher-quality rural bus services, substantial subsidies are needed. Secondly, the limited number of bus services is also a common concern among residents. Considering the cost of bus services and the travel habits of rural residents, it may be beneficial to increase the frequency of bus services in the morning to reduce peak-period waiting times. Thirdly, bus routes that do not travel directly periodic markets is a problem voiced by many villagers. According to Wang et al. (63), transfers significantly affect satisfaction with bus services. Given the limited destinations for rural residents’ travels, it may be worthwhile to consider designating important destinations as regular stops and adjusting bus routes to provide direct access to markets. Finally, for remote rural areas, the introduction of dedicated bus lines may be considered. Residents could pre-book their travel needs, and shuttle services could transport passengers directly from their homes to their destinations.

#### 5.2.3 Sensible site selection improves walkability

Due to distance considerations, rural residents seldom choose to walk to the markets. Walking not only promotes environmental sustainability but also contributes to the physical health of villagers. Therefore, it is essential to strategically plan the location of periodic markets in order to meet the commuting distance requirements of residents as far as is practicable. For residents of remote and scattered villages, options such as the dedicated bus lines, as mentioned in section 5.2.2 could serve as alternatives to walking.

In conclusion, in order to enhance the satisfaction of rural residents with regards to periodic market travel, and increase the vitality of markets, a number of recommendations are apparent: promote the mobility of residents while fostering low-carbon travel, improve the delineation of traffic functional zones and bus services. Such recommendations, however, would require robust government support.

## 6 Conclusion

Improving periodic market travel satisfaction is strongly linked to enhancing the quality of life and subjective well-being of rural Chinese residents. Travel satisfaction is closely associated with road environment and mode choice, yet the heterogeneity of road environment and periodic market travel satisfaction across different modes remains unresolved. In light of this, this study conducted field surveys in rural areas of Sichuan Province, China, and found that regardless of the mode of travel chosen, rural residents were satisfied with road cleanliness but dissatisfied with sidewalks and non-motorized vehicle lanes. Furthermore, this study focused on analyzing the heterogeneity of road environment factors influencing periodic market travel satisfaction across different modes. The results reveal that, except for road traffic disturbances and road deterioration, which did not significantly affect mode of travel, other factors proved significant. Specifically, (1) bus services affected overall satisfaction for pedestrians, non-motorized vehicles, and busses; (2) road cleanliness had a significant negative impact on non-motorized vehicle travel satisfaction; and, (3) under the mode of motorized vehicle travel, road greening and service facilities showed significant negative effects. Moreover, aspects of the road environment in need of urgent improvement for periodic market travel satisfaction were identified. Specifically, road functional zone delineation holds out tremendous potential for enhancing periodic market travel satisfaction among rural residents, while improving bus services would also contribute to enhancing periodic market travel satisfaction.

Though demographic factors and other travel-related variables such as travel time, frequency of travel, gender, age, vehicle ownership, and income level, are all associated with travel satisfaction, this study focused solely on examining the differences in satisfaction evaluation among different modes of travel. Although this study contributes to the literature, its findings are geographically limited to rural residents in Sichuan, China, and therefore, the results may not be wholly applicable to all rural residents in other countries. This study aims to discuss the impact of perceived road environments on market travel satisfaction without considering the role of objective road environments. Combining both subjective and objective road environments would enhance the persuasiveness of the research. The data collection for this study primarily relies on questionnaire surveys. In the future, the study will consider utilizing diverse datasets for analysis.

## Data availability statement

The original contributions presented in the study are included in the article/supplementary material, further inquiries can be directed to the corresponding author.

## Author contributions

HX: Conceptualization, Data curation, Formal analysis, Investigation, Methodology, Validation, Writing – original draft. PL: Resources, Writing – review & editing. HZ: Data curation, Methodology, Software, Visualization, Writing – review & editing. ML: Investigation, Validation, Writing – review & editing. HL: Resources, Writing – review & editing. IM: Writing – review & editing. YA: Funding acquisition, Project administration, Resources, Supervision, Writing – review & editing.
